# Imidazole–imidazolium picrate monohydrate

**DOI:** 10.1107/S160053681005169X

**Published:** 2010-12-15

**Authors:** Rodolfo Moreno-Fuquen, Regina De Almeida Santos, Lina Aguirre

**Affiliations:** a Departamento de Química - Facultad de Ciencias, Universidad del Valle, Apartado 25360, Santiago de Cali, Colombia; b Instituto de Química,IFSC, Universidade de São Paulo, São Carlos, Brazil; c Universidad Menendez Pelayo, Casa de la Ciencia, Pabellón del Perú, Avda Maria Luisa, s/n 41013, Sevilla, Spain

## Abstract

The asymmetric unit of the title compound, C_3_H_5_N_2_^+^·C_6_H_2_N_3_O_7_^−^·C_3_H_4_N_2_·H_2_O or H(C_3_H_4_N_2_)_2_^+^·C_6_H_2_N_3_O_7_^−^·H_2_O, contains a diimidazolium cationic unit, one picrate anion and one mol­ecule of water. In the crystal, the components are connected by N—H⋯O, N—H⋯N and O—H⋯O hydrogen bonds, forming a two-dimensional network parallel to (001). In addition, weak inter­molecular C—H⋯O hydrogen bonds lead to the formation of a three-dimensional network featuring *R*_5_^5^(19) rings.

## Related literature

For background to imidazolium salts see: Moreno-Fuquen *et al.* (2009*a* ▶,*b* ▶ ▶). For imidazole as an anti­fungal agent, see: Miranda *et al.* (1998 ▶); Rodriguez & Acosta (1997 ▶). For a description of the Cambridge Structural Database, see: Allen (2002 ▶). For hydrogen-bond geometries, see: Emsley (1984 ▶); Etter (1990 ▶); Nardelli (1995 ▶).
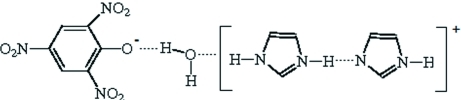


## Experimental

### 

#### Crystal data

C_3_H_5_N_2_^+^·C_6_H_2_N_3_O_7_^−^·C_3_H_4_N_2_·H_2_O
*M* *_r_* = 383.29
Orthorhombic, 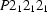

*a* = 3.8180 (1) Å
*b* = 20.8160 (8) Å
*c* = 21.4420 (8) Å
*V* = 1704.11 (10) Å^3^
*Z* = 4
Mo *K*α radiation
μ = 0.13 mm^−1^
*T* = 291 K
0.53 × 0.21 × 0.14 mm

#### Data collection

Bruker–Nonius KappaCCD diffractometer
12017 measured reflections
2207 independent reflections
1723 reflections with *I* > 2σ(*I*)
*R*_int_ = 0.062

#### Refinement

*R*[*F* ^2^ > 2σ(*F* ^2^)] = 0.040
*wR*(*F* ^2^) = 0.109
*S* = 1.06
2207 reflections
248 parameters
H-atom parameters constrained
Δρ_max_ = 0.14 e Å^−3^
Δρ_min_ = −0.15 e Å^−3^

### 

Data collection: *COLLECT* (Nonius, 2000 ▶); cell refinement: *SCALEPACK* (Otwinowski & Minor, 1997 ▶); data reduction: *DENZO* (Otwinowski & Minor, 1997 ▶) and *SCALEPACK*; program(s) used to solve structure: *SHELXS97* (Sheldrick, 2008 ▶); program(s) used to refine structure: *SHELXL97* (Sheldrick, 2008 ▶); molecular graphics: *ORTEP-3 for Windows* (Farrugia, 1997 ▶); software used to prepare material for publication: *WinGX* (Farrugia, 1999 ▶).

## Supplementary Material

Crystal structure: contains datablocks I, global. DOI: 10.1107/S160053681005169X/lh5175sup1.cif

Structure factors: contains datablocks I. DOI: 10.1107/S160053681005169X/lh5175Isup2.hkl

Additional supplementary materials:  crystallographic information; 3D view; checkCIF report

## Figures and Tables

**Table 1:** Hydrogen-bond geometry (Å, °)

*D*—H⋯*A*	*D*—H	H⋯*A*	*D*⋯*A*	*D*—H⋯*A*
N5—H5⋯N6	0.86	1.81	2.666 (3)	180
N4—H401⋯O5	0.94	1.78	2.714 (3)	176
O5—H501⋯O1	0.91	1.90	2.801 (3)	179
O5—H502⋯O1^i^	0.99	1.82	2.782 (3)	163
N7—H701⋯O1^ii^	0.88	2.01	2.876 (2)	167
C10—H101⋯O6^iii^	0.93	2.51	3.352 (4)	151
C9—H91⋯O8^iv^	0.93	2.58	3.481 (3)	162

Symmetry codes: (i) 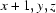; (ii) 
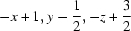
; (iii) 
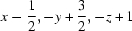
; (iv) 
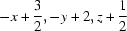
.

**Fig. 1.**
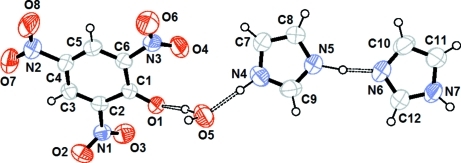
An ORTEP-3 (Farrugia, 1997) plot of the title compound with the atomic labelling scheme. Displacement ellipsoids are drawn at the 50% probability level. H atoms are shown as spheres of arbitary radius.

**Fig. 2.**
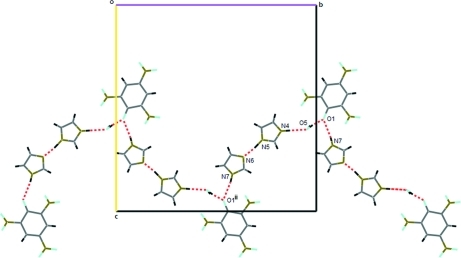
Part of the crystal structure of (I), showing the formation of chains of molecules running along the b axis. Symmetry code: (ii) -x + 1, y - 1/2, -z + 3/2.

**Fig. 3.**
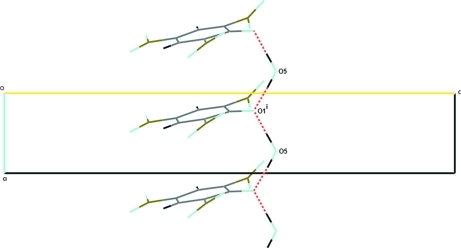
Part of the crystal structure of (I), showing the formation of chains of water molecules running along a axis. The O1 atom of the picrate ion, serves as a bridge in this chain. Symmetry code: (i) x + 1, y, z.

**Fig. 4.**
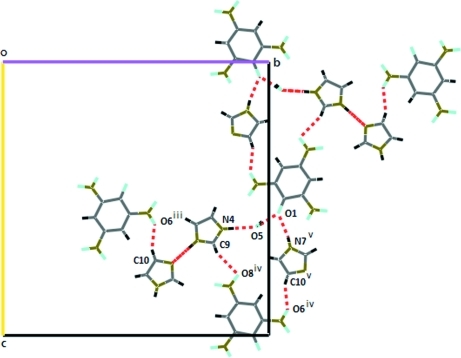
Part of the crystal structure of (I), showing the formation of R55(19) rings running along the c axis. Symmetry code: (iii) x - 1/2, -y + 3/2, -z + 1; (iv) -x + 3/2, -y + 2, z + 1/2; (v) i-x, y + 1/2, -z + 3/2.

## supplementary crystallographic information

### Comment

This work is part of a series of studies, related to the imidazole system which, has been conducted by the crystallography group at the University del Valle (Moreno-Fuquen *et al.*, 2009*a*,*b*). Imidazole, an aromatic heterocyclic, classified as an alkaloid, is present as an antifungal agent in commercial pharmaceutical products (Miranda *et al.*, 1998; Rodriguez & Acosta, 1997). A displacement ellipsoid plot of the title molecule (I) with the atomic numbering scheme is shown in Figure 1. The asymmetric unit contains two imidazole molecules as cationic unit, one picrate ion and one molecule of water. In the crystal,  molecules are connected by N—H···O, N—H···N and O—H···O hydrogen bonds. Interactions are of moderate character (Emsley, 1984) involving the following donor···acceptors: N5···N6, N4···O5, N7···O1 and O5···O1 and other weak C—H···O molecular interactions are also observed (Nardelli, 1995). In a substructure, the atom O5 in the molecule at (*x*, *y*, *z*) acts as donor and as an acceptor with atoms O1 and N4 in the molecule at (*x*, *y*, *z*). In addtion, atom N5 in the molecule at (*x*, *y*, *z*) acts as donor to the atom N6 in the molecule at (*x*, *y*, *z*). The atom N7 in the molecule at (*x*, *y*, *z*) acts as hydrogen bond donor to atom O1 in the molecule at (-*x* + 1, *y* - 1/2, -*z* + 3/2). These interactions form chains of molecules running along the *b* axis (see Fig. 2). In a second substructure, the atom O5 in the molecule at (*x*, *y*, *z*) acts as donor to atom O1 in the molecule at (*x* + 1, *y*, *z*), forming chains of water molecules running along *a* axis, where the atom O1 of the picrate ion, serves as a bridge in the chain (see Fig. 3). Finally, weak C10—H101···O6^iii^ and C9—H91···O8^iv^ interactions, together with the hydrogen bonds N4—H401···O5, O5—H501···O1 and N7—H701···O1^ii^, described above, form *R*_5_^5^(19) rings (Etter, 1990) which run along the *c* axis (see Fig. 4). The combination of these interactions allow the formation of three-dimensional network of the structure.

### Experimental

Reagents and solvents for the synthesis were obtained from the Aldrich Chemical Co., and were used without additional purification. The synthesis of the title compound was carried out by slow evaporation of a solution of imidazole (1.360 g. 0.02 mol) and picric acid (2.29 g. 0.01 mol) in 100 ml of dry acetonitrile. After a week, yellow prisms of a good quality suitable for X-ray analysis were obtained. *M*. p. 494 (1) K

### Refinement

In the Absence of significant anomalous dispersion effects the Friedel pairs were merged. The H atoms were located in a difference map, but were repositioned geometrically. They were initially refined with soft restraints on bond lengths and angles to regularize their geometry (C—H = 0.93, N—H5 = 0.86Å) and *U*_iso_(H) (1.2 times *U*_eq_ of the parent atom). After this, the positions were refined with riding constraints. Atoms H401, H501, H502 and H701 were found in a difference Fourier map and their coordinates were fixed with refined U_iso_(H) values.

### Crystal data

**Table:**

C_3_H_5_N_2_^+^·C_6_H_2_N_3_O_7_^−^·C_3_H_4_N_2_·H_2_O	*D*_x_ = 1.494 Mg m^−^^3^
*M**_r_* = 383.29	Melting point: 494(1) K
Orthorhombic, *P*2_1_2_1_2_1_	Mo *K*α radiation, λ = 0.71073 Å
Hall symbol: P 2ac 2ab	Cell parameters from 6888 reflections
*a* = 3.8180 (1) Å	θ = 2.9–27.1°
*b* = 20.8160 (8) Å	µ = 0.13 mm^−^^1^
*c* = 21.4420 (8) Å	*T* = 291 K
*V* = 1704.11 (10) Å^3^	Prism, yellow
*Z* = 4	0.53 × 0.21 × 0.14 mm
*F*(000) = 792	

### Data collection

**Table:**

Bruker–Nonius KappaCCD diffractometer	1723 reflections with *I* > 2σ(*I*)
Radiation source: fine-focus sealed tube	*R*_int_ = 0.062
horizonally mounted graphite crystal	θ_max_ = 27.1°, θ_min_ = 3.0°
Detector resolution: 9 pixels mm^-1^	*h* = −4→3
CCD scans	*k* = −20→26
12017 measured reflections	*l* = −27→26
2207 independent reflections	

### Refinement

**Table:**

Refinement on *F*^2^	Primary atom site location: structure-invariant direct methods
Least-squares matrix: full	Secondary atom site location: difference Fourier map
*R*[*F*^2^ > 2σ(*F*^2^)] = 0.040	Hydrogen site location: inferred from neighbouring sites
*wR*(*F*^2^) = 0.109	H-atom parameters constrained
*S* = 1.06	*w* = 1/[σ^2^(*F*_o_^2^) + (0.0567*P*)^2^ + 0.1542*P*] where *P* = (*F*_o_^2^ + 2*F*_c_^2^)/3
2207 reflections	(Δ/σ)_max_ < 0.001
248 parameters	Δρ_max_ = 0.14 e Å^−^^3^
0 restraints	Δρ_min_ = −0.15 e Å^−^^3^

### Special details

**Table:**

Geometry. All e.s.d.'s (except the e.s.d. in the dihedral angle between two l.s. planes) are estimated using the full covariance matrix. The cell e.s.d.'s are taken into account individually in the estimation of e.s.d.'s in distances, angles and torsion angles; correlations between e.s.d.'s in cell parameters are only used when they are defined by crystal symmetry. An approximate (isotropic) treatment of cell e.s.d.'s is used for estimating e.s.d.'s involving l.s. planes.
Refinement. Refinement of *F*^2^ against ALL reflections. The weighted *R*-factor *wR* and goodness of fit *S* are based on *F*^2^, conventional *R*-factors *R* are based on *F*, with *F* set to zero for negative *F*^2^. The threshold expression of *F*^2^ > σ(*F*^2^) is used only for calculating *R*-factors(gt) *etc*. and is not relevant to the choice of reflections for refinement. *R*-factors based on *F*^2^ are statistically about twice as large as those based on *F*, and *R*- factors based on ALL data will be even larger.

### Fractional atomic coordinates and isotropic or equivalent isotropic displacement parameters (Å^2^)

**Table:**

	*x*	*y*	*z*	*U*_iso_*/*U*_eq_	
O1	0.2191 (5)	1.03522 (7)	0.55396 (7)	0.0571 (4)	
O2	−0.1566 (6)	1.14493 (9)	0.57670 (8)	0.0731 (5)	
O3	0.1895 (7)	1.21912 (8)	0.54543 (9)	0.0788 (6)	
O4	0.2390 (9)	0.92426 (8)	0.48892 (9)	0.0914 (8)	
O5	0.7228 (7)	0.95183 (9)	0.60361 (10)	0.0802 (6)	
O6	0.5569 (8)	0.92982 (10)	0.40721 (10)	0.0965 (8)	
O7	0.2812 (11)	1.20386 (11)	0.31429 (10)	0.1096 (10)	
O8	0.4859 (10)	1.11533 (12)	0.27852 (9)	0.1128 (11)	
N1	0.0590 (5)	1.16612 (9)	0.54037 (9)	0.0515 (5)	
N2	0.3639 (9)	1.14743 (12)	0.32037 (10)	0.0774 (7)	
N3	0.3776 (7)	0.95481 (9)	0.44756 (9)	0.0622 (6)	
N4	0.5815 (7)	0.82430 (10)	0.61111 (11)	0.0659 (6)	
N5	0.5551 (7)	0.73058 (10)	0.65339 (10)	0.0660 (6)	
H5	0.5742	0.6999	0.6800	0.079*	
N6	0.6123 (7)	0.63572 (11)	0.73624 (9)	0.0693 (6)	
N7	0.7046 (8)	0.58105 (11)	0.82023 (9)	0.0694 (7)	
C1	0.2292 (6)	1.05863 (10)	0.50031 (9)	0.0464 (5)	
C2	0.1650 (6)	1.12594 (10)	0.48771 (9)	0.0460 (5)	
C3	0.2078 (7)	1.15485 (10)	0.43161 (10)	0.0523 (6)	
H31	0.1686	1.1987	0.4270	0.063*	
C4	0.3112 (8)	1.11772 (11)	0.38114 (10)	0.0540 (6)	
C5	0.3698 (7)	1.05261 (11)	0.38734 (10)	0.0530 (6)	
H51	0.4405	1.0284	0.3532	0.064*	
C6	0.3229 (7)	1.02395 (10)	0.44446 (10)	0.0496 (5)	
C7	0.4412 (8)	0.78433 (12)	0.56804 (12)	0.0636 (7)	
H71	0.3711	0.7952	0.5279	0.076*	
C8	0.4228 (8)	0.72631 (12)	0.59449 (12)	0.0648 (7)	
H81	0.3348	0.6893	0.5759	0.078*	
C9	0.6470 (9)	0.79029 (14)	0.66155 (12)	0.0693 (7)	
H91	0.7453	0.8066	0.6979	0.083*	
C10	0.5174 (9)	0.57373 (15)	0.72587 (12)	0.0726 (8)	
H101	0.4286	0.5577	0.6886	0.087*	
C11	0.5717 (10)	0.53967 (15)	0.77749 (12)	0.0766 (9)	
H111	0.5274	0.4961	0.7831	0.092*	
C12	0.7269 (10)	0.63802 (14)	0.79433 (12)	0.0729 (8)	
H121	0.8111	0.6747	0.8140	0.088*	
H401	0.6413	0.8680	0.6086	0.095 (10)*	
H701	0.7523	0.5718	0.8593	0.084 (9)*	
H501	0.5605	0.9792	0.5877	0.41 (7)*	
H502	0.9212	0.9740	0.5833	0.17 (2)*	

### Atomic displacement parameters (Å^2^)

**Table:**

	*U*^11^	*U*^22^	*U*^33^	*U*^12^	*U*^13^	*U*^23^
O1	0.0768 (12)	0.0511 (8)	0.0435 (7)	−0.0053 (9)	0.0018 (8)	0.0016 (6)
O2	0.0723 (12)	0.0860 (13)	0.0609 (10)	0.0056 (12)	0.0146 (11)	−0.0090 (10)
O3	0.0998 (15)	0.0529 (9)	0.0836 (12)	0.0004 (11)	−0.0011 (13)	−0.0191 (9)
O4	0.152 (2)	0.0493 (9)	0.0731 (12)	−0.0071 (13)	0.0180 (16)	0.0000 (9)
O5	0.0931 (16)	0.0565 (10)	0.0909 (13)	−0.0093 (11)	0.0083 (14)	0.0037 (9)
O6	0.142 (2)	0.0693 (11)	0.0781 (12)	0.0391 (14)	0.0226 (16)	−0.0082 (10)
O7	0.183 (3)	0.0739 (13)	0.0718 (12)	0.0005 (19)	0.0005 (19)	0.0242 (11)
O8	0.180 (3)	0.1017 (16)	0.0570 (10)	−0.005 (2)	0.0428 (17)	0.0030 (12)
N1	0.0532 (11)	0.0501 (10)	0.0514 (10)	0.0066 (9)	−0.0063 (10)	−0.0048 (9)
N2	0.108 (2)	0.0727 (14)	0.0519 (12)	−0.0131 (16)	0.0059 (14)	0.0083 (11)
N3	0.0874 (16)	0.0495 (10)	0.0498 (10)	0.0086 (12)	−0.0034 (12)	−0.0073 (9)
N4	0.0724 (15)	0.0513 (11)	0.0742 (13)	−0.0013 (11)	0.0062 (12)	−0.0015 (10)
N5	0.0846 (16)	0.0611 (12)	0.0525 (11)	−0.0011 (12)	−0.0043 (12)	0.0073 (10)
N6	0.0808 (16)	0.0752 (14)	0.0520 (11)	0.0021 (14)	−0.0064 (12)	0.0104 (10)
N7	0.0958 (18)	0.0699 (13)	0.0425 (10)	0.0095 (14)	−0.0023 (12)	0.0080 (10)
C1	0.0505 (13)	0.0450 (10)	0.0437 (11)	−0.0041 (10)	−0.0014 (10)	−0.0019 (9)
C2	0.0462 (12)	0.0459 (10)	0.0460 (10)	0.0010 (10)	−0.0026 (10)	−0.0058 (9)
C3	0.0582 (14)	0.0442 (11)	0.0545 (12)	0.0004 (11)	−0.0052 (12)	0.0008 (10)
C4	0.0626 (15)	0.0556 (12)	0.0439 (11)	−0.0069 (12)	−0.0005 (11)	0.0059 (10)
C5	0.0580 (14)	0.0578 (12)	0.0433 (11)	0.0015 (12)	0.0029 (11)	−0.0063 (10)
C6	0.0570 (14)	0.0437 (11)	0.0481 (11)	0.0008 (11)	−0.0022 (11)	−0.0034 (9)
C7	0.0769 (19)	0.0611 (14)	0.0529 (13)	0.0032 (14)	−0.0062 (13)	0.0025 (11)
C8	0.0818 (19)	0.0564 (13)	0.0562 (13)	−0.0061 (14)	−0.0071 (14)	−0.0028 (11)
C9	0.0819 (19)	0.0720 (16)	0.0541 (14)	−0.0056 (16)	−0.0044 (15)	−0.0119 (13)
C10	0.084 (2)	0.0856 (18)	0.0482 (13)	−0.0080 (18)	−0.0031 (15)	−0.0003 (13)
C11	0.104 (3)	0.0689 (15)	0.0567 (14)	−0.0112 (18)	0.0035 (16)	0.0030 (13)
C12	0.098 (2)	0.0665 (15)	0.0542 (13)	0.0016 (17)	−0.0076 (16)	0.0045 (12)

### Geometric parameters (Å, °)

**Table:**

O1—C1	1.250 (2)	N7—C12	1.312 (3)
O2—N1	1.216 (3)	N7—C11	1.356 (4)
O3—N1	1.216 (3)	N7—H701	0.880
O4—N3	1.213 (3)	C1—C6	1.443 (3)
O5—H501	0.908	C1—C2	1.448 (3)
O5—H502	0.988	C2—C3	1.355 (3)
O6—N3	1.220 (3)	C3—C4	1.387 (3)
O7—N2	1.223 (3)	C3—H31	0.9300
O8—N2	1.212 (3)	C4—C5	1.380 (3)
N1—C2	1.462 (3)	C5—C6	1.374 (3)
N2—C4	1.456 (3)	C5—H51	0.9300
N3—C6	1.456 (3)	C7—C8	1.336 (4)
N4—C9	1.317 (3)	C7—H71	0.9300
N4—C7	1.354 (3)	C8—H81	0.9300
N4—H401	0.940	C9—H91	0.9300
N5—C9	1.303 (3)	C10—C11	1.331 (4)
N5—C8	1.363 (3)	C10—H101	0.9300
N5—H5	0.8600	C11—H111	0.9300
N6—C12	1.321 (3)	C12—H121	0.9300
N6—C10	1.359 (4)		
			
H501—O5—H502	93.71	C4—C3—H31	120.7
O3—N1—O2	123.3 (2)	C5—C4—C3	121.2 (2)
O3—N1—C2	118.3 (2)	C5—C4—N2	118.7 (2)
O2—N1—C2	118.34 (19)	C3—C4—N2	120.0 (2)
O8—N2—O7	123.4 (2)	C6—C5—C4	119.4 (2)
O8—N2—C4	118.8 (2)	C6—C5—H51	120.3
O7—N2—C4	117.9 (3)	C4—C5—H51	120.3
O4—N3—O6	122.7 (2)	C5—C6—C1	123.69 (19)
O4—N3—C6	119.3 (2)	C5—C6—N3	116.8 (2)
O6—N3—C6	118.0 (2)	C1—C6—N3	119.48 (19)
C9—N4—C7	107.8 (2)	C8—C7—N4	106.7 (2)
C9—N4—H401	121.4	C8—C7—H71	126.7
C7—N4—H401	130.8	N4—C7—H71	126.7
C9—N5—C8	106.6 (2)	C7—C8—N5	108.4 (2)
C9—N5—H5	126.7	C7—C8—H81	125.8
C8—N5—H5	126.7	N5—C8—H81	125.8
C12—N6—C10	106.1 (2)	N5—C9—N4	110.6 (2)
C12—N7—C11	108.2 (2)	N5—C9—H91	124.7
C12—N7—H701	126.0	N4—C9—H91	124.7
C11—N7—H701	125.6	C11—C10—N6	109.2 (2)
O1—C1—C6	125.19 (19)	C11—C10—H101	125.4
O1—C1—C2	122.94 (19)	N6—C10—H101	125.4
C6—C1—C2	111.79 (17)	C10—C11—N7	106.4 (3)
C3—C2—C1	125.10 (19)	C10—C11—H111	126.8
C3—C2—N1	117.70 (18)	N7—C11—H111	126.8
C1—C2—N1	117.15 (17)	N7—C12—N6	110.2 (3)
C2—C3—C4	118.6 (2)	N7—C12—H121	124.9
C2—C3—H31	120.7	N6—C12—H121	124.9
			
O1—C1—C2—C3	172.9 (3)	C4—C5—C6—N3	178.0 (3)
C6—C1—C2—C3	−4.0 (4)	O1—C1—C6—C5	−172.4 (3)
O1—C1—C2—N1	−4.6 (4)	C2—C1—C6—C5	4.4 (4)
C6—C1—C2—N1	178.5 (2)	O1—C1—C6—N3	6.7 (4)
O3—N1—C2—C3	−40.4 (3)	C2—C1—C6—N3	−176.5 (2)
O2—N1—C2—C3	139.3 (2)	O4—N3—C6—C5	−156.8 (3)
O3—N1—C2—C1	137.3 (2)	O6—N3—C6—C5	21.7 (4)
O2—N1—C2—C1	−43.0 (3)	O4—N3—C6—C1	24.0 (4)
C1—C2—C3—C4	2.0 (4)	O6—N3—C6—C1	−157.5 (3)
N1—C2—C3—C4	179.5 (2)	C9—N4—C7—C8	0.6 (3)
C2—C3—C4—C5	0.1 (4)	N4—C7—C8—N5	−0.6 (3)
C2—C3—C4—N2	−179.2 (3)	C9—N5—C8—C7	0.5 (4)
O8—N2—C4—C5	−6.2 (5)	C8—N5—C9—N4	−0.1 (4)
O7—N2—C4—C5	173.6 (3)	C7—N4—C9—N5	−0.3 (4)
O8—N2—C4—C3	173.1 (3)	C12—N6—C10—C11	0.6 (4)
O7—N2—C4—C3	−7.1 (5)	N6—C10—C11—N7	−0.5 (4)
C3—C4—C5—C6	0.4 (4)	C12—N7—C11—C10	0.1 (4)
N2—C4—C5—C6	179.7 (3)	C11—N7—C12—N6	0.2 (4)
C4—C5—C6—C1	−2.9 (4)	C10—N6—C12—N7	−0.5 (4)

### Hydrogen-bond geometry (Å, °)

**Table:**

*D*—H···*A*	*D*—H	H···*A*	*D*···*A*	*D*—H···*A*
N5—H5···N6	0.86	1.81	2.666 (3)	180
N4—H401···O5	0.94	1.78	2.714 (3)	176
O5—H501···O1	0.91	1.90	2.801 (3)	179
O5—H502···O1^i^	0.99	1.82	2.782 (3)	163
N7—H701···O1^ii^	0.88	2.01	2.876 (2)	167
C10—H101···O6^iii^	0.93	2.51	3.352 (4)	151
C9—H91···O8^iv^	0.93	2.58	3.481 (3)	162

Symmetry codes:  (i) *x*+1, *y*, *z*; (ii) −*x*+1, *y*−1/2, −*z*+3/2; (iii) *x*−1/2, −*y*+3/2, −*z*+1; (iv) −*x*+3/2, −*y*+2, *z*+1/2.
